# Genotypic and phenotypic diversity of *Lactobacillus rhamnosus* clinical isolates, their comparison with strain GG and their recognition by complement system

**DOI:** 10.1371/journal.pone.0176739

**Published:** 2017-05-11

**Authors:** Eija Nissilä, François P. Douillard, Jarmo Ritari, Lars Paulin, Hanna M. Järvinen, Pia Rasinkangas, Karita Haapasalo, Seppo Meri, Hanna Jarva, Willem M. de Vos

**Affiliations:** 1 Department of Bacteriology & Immunology and Research Programs Unit, Immunobiology, University of Helsinki, Finland; 2 Department of Veterinary Biosciences, Division of Microbiology and Epidemiology, University of Helsinki, Helsinki, Finland; 3 Finnish Red Cross Blood Service, Helsinki, Finland; 4 Institute of Biotechnology, University of Helsinki, Helsinki, Finland; 5 Helsinki University Hospital Laboratory HUSLAB, Helsinki, Finland; 6 Laboratory of Microbiology, Wageningen University, Wageningen, The Netherlands; University of Kansas Medical Center, UNITED STATES

## Abstract

*Lactobacillus rhamnosus* strains are ubiquitous in fermented foods, and in the human body where they are commensals naturally present in the normal microbiota composition of gut, vagina and skin. However, in some cases, *Lactobacillus* spp. have been implicated in bacteremia. The aim of the study was to examine the genomic and immunological properties of 16 clinical blood isolates of *L*. *rhamnosus* and to compare them to the well-studied *L*. *rhamnosus* probiotic strain GG. Blood cultures from bacteremic patients were collected at the Helsinki University Hospital laboratory in 2005–2011 and *L*. *rhamnosus* strains were isolated and characterized by genomic sequencing. The capacity of the *L*. *rhamnosus* strains to activate serum complement was studied using immunological assays for complement factor C3a and the terminal pathway complement complex (TCC). Binding of complement regulators factor H and C4bp was also determined using radioligand assays. Furthermore, the isolated strains were evaluated for their ability to aggregate platelets and to form biofilms *in vitro*. Genomic comparison between the clinical *L*. *rhamnosus* strains showed them to be clearly different from *L*. *rhamnosus* GG and to cluster in two distinct lineages. All *L*. *rhamnosus* strains activated complement in serum and none of them bound complement regulators. Four out of 16 clinical blood isolates induced platelet aggregation and/or formed more biofilms than *L*. *rhamnosus* GG, which did not display platelet aggregation activity nor showed strong biofilm formation. These findings suggest that clinical *L*. *rhamnosus* isolates show considerable heterogeneity but are clearly different from *L*. *rhamnosus* GG at the genomic level. All *L*. *rhamnosus* strains are still normally recognized by the human complement system.

## Introduction

Lactobacilli are Gram-positive, microaerophilic, or facultative anaerobic rods [1] that are commonly distributed in the environment, animals and humans [2]. In human, lactobacilli live as commensals in the oral, gastrointestinal and female genital tracts [3]. An isolate from the human intestinal tract, *Lactobacillus rhamnosus* strain GG (ATCC 53103) is a widely used and well-characterized probiotic strain [4] which has been suggested to have health-promoting effects including the prevention and treatment of gastro-intestinal infections and diarrhea and stimulation of immune responses to prevent allergic symptoms [5].

Although no virulence traits have been described for Lactobacilli and their safety for use in food products has been established [6], several Lactobacillus spp. have been isolated from infected tissues, such as peritonitis [7, 8], endocarditis [9], pneumonia [10], deep abdominal abscesses [11] and blood [9, 12]. The most commonly isolated Lactobacillus spp. from bacteremia include *L*. *rhamnosus* and *L*. *casei*, which are often used as probiotics [9]. Risk factors associated with *Lactobacillus* bacteremia consist of weakened host defences and serious underlying disease [13]. The symptoms of clinical study cases suffering from Lactobacillus-associated bacteremia can vary from asymptomatic to severe septicemia [9]. It has raised a question whether these isolates have developed any properties found from pathogenic bacteria such as group A streptococci (*S*. *pyogenes*), which have evolved multiple mechanisms to adapt, survive and grow in the human body [14]. Some of these features have been characterized in studies with different Lactobacillus strains isolated from blood or infected tissues. *L*. *rhamnosus* and *L*. *salivarius* strains but not *L*. *rhamnosus* GG have been found to induce platelet aggregation [15–19]. *L*. *rhamnosus* strains isolated from blood have been shown to have differences in binding to mucus, collagen and fibrinogen and in survival in serum when compared to *L*. *rhamnosus* GG [20]. Two *Lactobacillus rhamnosus* clinical isolates from dental pulp infection were found to have a modified exopolysaccharide (EPS) cluster which was different from *L*. *rhamnosus* GG [21].

It has not been demonstrated unequivocally whether the isolated *L*. *rhamnosus* strains from infected tissue or blood of bacteremic patients were similar to *L*. *rhamnosus* GG at genomic level and whether they have similar or distinct features with *L*. *rhamnosus* GG in terms of immunological and immunogenic properties in the human host. This has now become possible since the complete genome sequence of *L*. *rhamnosus* GG has been determined [22]. Hence, in the present study, we have isolated and sequenced 4 new *L*. *rhamnosus* strains from blood cultures of bacteremic patients and performed genetic and phenotypic characterization of these and 12 earlier described strains [23] collected during years 2005–2011 in Helsinki University Hospital laboratory (HUSLAB). First, we explored how different or similar these strains are from the widely consumed probiotic strain *L*. *rhamnosus* GG at genomic level. Secondly, we investigated the phenotypic features of these strains *in vitro* and compared them to *L*. *rhamnosus* GG and selected Gram-positive pathogenic strains. Specifically, we examined their ability to activate or inhibit complement system, induce platelet aggregation and form biofilms. Finally, we investigated the possible associations between their phenotypic features and surface proteins and exopolysaccharide associated proteins that are most likely to interact with the environment and the host.

Our study revealed that all *L*. *rhamnosus* blood isolates were clearly different from *L*. *rhamnosus* GG as well as each other at the genome level and clustered in two groups. All *L*. *rhamnosus* strains induced complement activation in serum when formation of C3a and TCC were measured and none of them bound the complement inhibitors factor H (FH) and C4bp. Interestingly, four out of 16 strains were able to induce platelet aggregation and one of those and three other strains also formed a stronger biofilm compared to *L*. *rhamnosus* GG. Clear differences were seen in the organization and the presence of genes encoding surface polysaccharides, including EPS/capsular polysaccharide (CPS), between the clinical strains belonging to the so-called cluster A and cluster B.

## Materials and methods

### Ethics statement

All persons who donated blood for this study provided a written informed consent. The study protocol has been approved by the Academy of Finland (Projects 28 137389, 141140 and 1272870).

### Bacterial isolates and growth conditions

*Lactobacillus* strains used in the study are listed in Table 1 and includes 4 newly sequenced strains and 12 strains previously published [22, 23]. Clinical blood isolates of *L*. *rhamnosus* strains were obtained from HUSLAB and collected from bacteremic patients between years 2005 and 2011. *L*. *rhamnosus GG* (ATCC 53103) was obtained from the Valio culture collection (Valio Ltd., Helsinki, Finland). *L*. *salivarius* CCUG 47825 was a kind gift from Prof. Paul W. O’Toole (University College Cork, Cork, Ireland) [19]. Pathogenic control *Streptococcus pyogenes* strain st369 was obtained from the collection of the National Institute for Health and Welfare (Prof. Jaana Vuopio) and emm8 from ATCC (ATCC catalogue number 12349). *S*. *pyogenes* strains were used as controls for complement activity and FH and C4bp binding assays [24]. Lactobacilli were cultured at 37°C in de Man-Rogosa-Sharpe (MRS) medium (Difco BD, NJ, USA). *S*. *pyogenes* strains were grown at 37°C with 5% CO_2_ on blood-agar plates supplemented with colistin and oxolinic acid or in Todd-Hewitt broth (Difco BD, NJ, USA). For *in vitro* assays *Lactobacillus* strains were cultured at 37°C in MRS medium overnight. The overnight bacterial cultures were diluted 1:100 and cultured until the OD_600_ reached 0.5–0.7 for log phase and overnight for stationary phase. The cells were collected by centrifugation and glycerol was added until 20% to freeze the cultures. The strains were stored at -70°C.

**Table 1:** Genetic information of *L*. *rhamnosus* strains isolated from blood cultures in HUSLAB, 2005–2011.

Strain name	Plasmid-matching* contigs	G+C (%)	ORFs
*L*. *rhamnosus* GG (ATCC 53103)	0	0	0
Lrh7	0	0	0
Lrh8	0	0	0
Lrh9	0	0	0
Lrh11	0	0	0
Lrh13	1	44.4	14
Lrh14	0	0	0
Lrh18	0	0	0
Lrh20	0	0	0
Lrh22	1	44.7	63
Lrh28	1	44.2	34
Lrh30	3	43.2	23
Lrh32	0	0	0
Lrh38	0	0	0
Lrh39	0	0	0
Lrh46	0	0	0
Lrh47	2	43.5	42

ORF indicates open reading frame.

### Genome sequencing

DNA was extracted from the bacterial cultures as described previously [23]. Libraries from genomic DNA from *L*. *rhamnosus* strains were done for both 454 sequencing and MiSeq sequencing following recommendation from respective manufacturer. The 454 libraries were sequenced using a Genome Sequencer FLX (Roche) and titanium chemistry with read lengths of ca 400 bp. The Illumina libraries were paired-end sequenced (250 bp + 250 bp) using a MISeq (Illumina). All library preparations and sequencing were done at the DNA sequencing and genomics laboratory, Institute Biotechnology, University of Helsinki, Finland. A combined 454 and MiSeq read assembly were performed using the Celera Assembler v6.1 (http://sourceforge.net/apps/mediawiki/wgs-assembler/index.php?title=Main_Page). Genomes were annotated using the RAST server [25]. The new genome sequences were deposited to public depository databases but most of them were published earlier by Ceapa *et al*. [23] or by others (see for details Table 1).

### Bioinformatics analysis

The orthology prediction was performed using sequence clustering and search program Usearch v8.0.1623 [26]. To define orthologous groups, all the predicted ORF amino acid sequences of all the strains were first ordered by length using the *sortbylength* command, and then clustered with the *cluster_smallmem* command using identity threshold of 0.9. Thus, the members of each ORF sequence cluster shared at least 90% amino acid identity over their whole sequence lengths. The longest sequence in each cluster was chosen as the representative sequence of that orthologous group. The pangenome was defined as the set of representative sequences of the orthologous groups. The ortholog data was managed using R v. 3.2.2 (R) with Biostrings library v. 2.40.2 [27] and in-house scripts. The ortholog copy number data was used for hierarchical clustering of the strains by first removing orthologs that had variance <0.06 over the strains, and then calculating Euclidean distances between the strains and applying Ward's minimum variance method to the distances using R functions *dist* and *hclust*, respectively. Clustering dendrograms were drawn with R library dendextend v. 1.3.0 [28], and the function *heatmap*.*2* from R library gplots v. 3.0.1. [29] was used for producing a row-wise centered and scaled heatmap of the filtered ortholog number matrix. Strains were compared at nucleotide level to the *L*. *rhamnosus* GG genome using blastn program v. 2.2.31+ with parameters *-task magablast*, *-evalue 0*.*001* and *-perc_identity 95*. Mean similarity of aligned fragments over 10 kb was calculated for each strain.

### Complement activity determination using C3a and TCC ELISA

The frozen batches of log phase bacteria were used to activate complement in the serum pool of healthy volunteers. Bacterial cells were washed with PBS and adjusted to have 10^7^ bacterial cells in 100 μl of PBS. The bacteria were mixed with the same volume of serum and incubated for 30 minutes at 37°C in agitation at 500 rpm. The reactions were stopped by adding EDTA to a final concentration of 15 mM and by transferring the samples on ice. Cells were separated from serum samples by centrifugation at 5,900g for 3 minutes and stored at -70°C for C3a and SC5b-9 (TCC) analysis. Complement C3a and SC5b-9 were analyzed from the samples using the commercial MicroVue C3a EIA (Quidel, Tecomedical) and ELISA for TCC determination as described earlier [30]. For C3a measurements serum samples were diluted 1 to 10,000 and for TCC assay 1 to 2,000.

### Direct ^125^I-factor H and C4bp binding assays

The binding of FH and C4bp were performed as described earlier [31]. Shortly, FH and C4bp were labelled with iodine (^125^I; Perkin Elmer) using the Iodogen method [32]. The specific activities of the labelled proteins were 1–2 x 10^7^ cpm/μg. Each bacterial strain (3 × 10^7^ log phase bacteria/reaction) was incubated with 25,000 cpm of radiolabeled FH or C4bp in 100 μl of gelatin-Veronal buffered saline for 30 minutes at 37°C. The reaction mixtures were centrifuged (at 10,000g for 3 minutes) through 20% sucrose, and the radioactivity of each pellet and supernatant was measured with gamma counter. *S*. *pyogenes* strains were used as a negative (emm8) and positive (st369) controls for FH binding and positive control for C4bp binding [33]. The ratios of bound (pellet) to total activity (pellet + supernatant) were calculated as percentage.

### Platelet preparation and aggregometry

Blood samples were collected from healthy donors as previously described [34]. Nine ml of blood was collected into 3.2% Na-citrate tubes (Vacuette, ref. 455322, Greiner-bio-one) via venipuncture from a cubital vein from a healthy donor using 20G needle. Whole blood was centrifuged at 180g for 12 minutes at 22°C without using brake. Platelet rich plasma (PRP) was collected slowly above the buffy coat with a plastic Pasteur pipette into separate plastic tube. The remaining blood was mixed and centrifuged again at 2,000g for 10 minutes at 22°C to obtain platelet poor plasma (PPP). The platelet count was measured with Sysmex XK-21 cell counter and adjusted with PPP to the desired concentration.

Platelet aggregation was carried out in a profiler aggregometer (PAP-4, Bio/Data corp.), with light transmission through PPP presenting 100% aggregation and that through PRP representing 0% aggregation. PRP was used in a test well and the change in transmission over time was a signal of the platelet aggregation. The frozen batches of bacterial cells were washed with PBS three times. The final volume was adjusted to get 2 x 10^9^ cfu/ml in PBS for platelet aggregation experiments. *Lactobacillus* strains were determined for their ability to induce platelet aggregation by the addition of 25 μl of a bacterial suspension to 475 μl of PRP. One minute after agonist ADP or bacteria was added into the well the platelet response was monitored for a maximum 30 minutes. Platelet viability was confirmed by addition to PRP of agonist adenosine diphosphate (ADP) to a final concentration of 5 μM.

### Biofilm assay

The microtiter plate biofilm assay is a static assay that has been used for examining biofilm formation [35]. The wells of 24-well cell culture plate (Greiner Bio-One, Germany) were filled in with serial dilution (1 to 10) of an overnight bacterial culture. After an incubation for 18 hour at 37°C, the wells were washed with aqua and stained with crystal violet. The crystal violet stained plates were dried and scanned with Odyssey IR scanner (Licor) at 700 nm channel and integrated intensities analyzed using Odyssey (v 2.1) analysis software. The bound dye was also solubilized in 250 μl of 70% ethanol, transferred into 96-well plate and the optical density of the ethanol-dye solution was determined at 600 nm using a microplate reader (Fluostar Optima, BMG Labtech, Germany). The correlation between the methods was high (97.3%) indicating that the crystal violet was a strong fluorophore at 700 nm and Odyssey scanner is a useful tool for determining biofilms.

### Western blot analysis of cell wall proteins

Cell wall proteins were extracted and the presence of SpaCBA and SpaFED pili were determined using Western Blot analysis as earlier described [22].

### Statistical analysis

All values are presented as a mean ± SD. Where applicable, a 2-tailed Student's t-test was used to analyse the differences between groups (Excel software; Microsoft, Redmond, WA, USA).

## Results

### Genomic features and organization of *L*. *rhamnosus* clinical blood isolates vs. *L*. *rhamnosus* GG

All *L*. *rhamnosus* strains described in the present study were isolated from clinical blood cultures collected during 2005–2011 from the Helsinki University Hospital laboratory. The genomes of four strains are reported here while that of the other 12 have been described earlier [23]. Their general genomic information was compared to that of *L*. *rhamnosus* GG (Table 1 and Table 2). Genome size, G+C content and open reading frame (ORF) count of annotated protein coding sequences varied greatly between the strains and differed from *L*. *rhamnosus* GG. The genome sizes ranged from 2.86 to 3.17 Mb. The average genome size was 3.0 Mb ± 0.16 Mb which is in the same range as other members of the *L*. *rhamnosus* species [23]. The G+C content varied among the genomes from 46.6 to 46.8% being closely similar to *L*. *rhamnosus* GG (46.7%) while the number of ORF predictions varied between 2648 and 3010, exceeding that of *L*. *rhamnosus* GG (2926).

**Table 2:** Plasmids of the *L*. *rhamnosus* strains.

Strain name	Plasmid-matching*contigs	G+C (%)	ORFs
*L*. *rhamnosus* GG (ATCC 53103)	0	0	0
Lrh7	0	0	0
Lrh8	0	0	0
Lrh9	0	0	0
Lrh11	0	0	0
Lrh13	1	44.4	14
Lrh14	0	0	0
Lrh18	0	0	0
Lrh20	0	0	0
Lrh22	1	44.7	63
Lrh28	1	44.2	34
Lrh30	3	43.2	23
Lrh32	0	0	0
Lrh38	0	0	0
Lrh39	0	0	0
Lrh46	0	0	0
Lrh47	2	43.5	42

* indicates BLASTN e-value < 0.000001, identity > 85%, alignment length > 5kb; ORF indicates open reading frame

**Fig 1:**
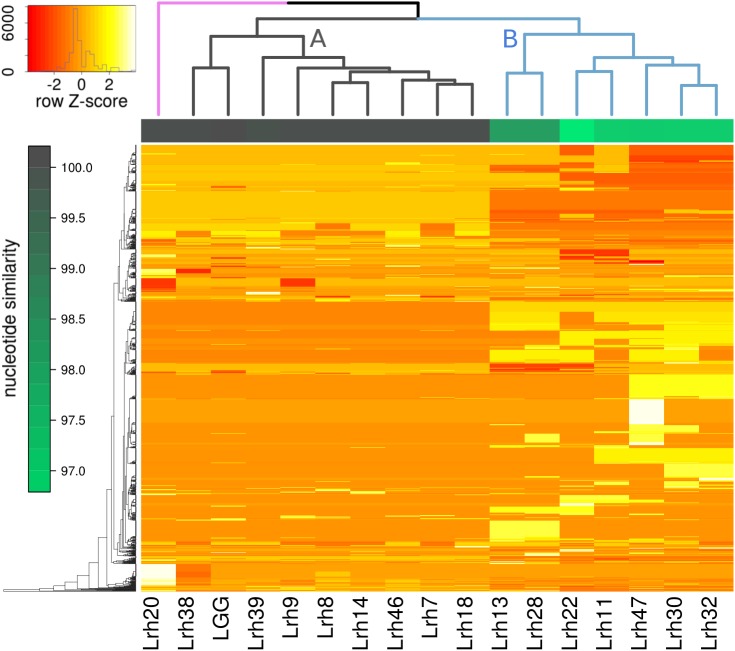
Clustering analysis of strains by ortholog copy numbers. The heat map rows represent centered and scaled ortholog copy numbers found in the strains. The dendrogram on the columns shows hierarchical clustering of the strains based on their ortholog copy numbers. Columns are annotated by each strain's BLAST nucleotide similarity to the *L*. *rhamnosus* GG genome.

To analyze the similarity of the strains against *L*. *rhamnosus* GG at sequence level, a dendrogram based on cluster analysis of the presence of orthologs at 90% sequence identity was created (Fig 1). This showed that the strains could be grouped into two main clusters and one separate strain (Lrh20). It shows that the *L*. *rhamnosus* isolates are different from each other at the sequence level and more remarkably, all are very different at the genomic level from *L*. *rhamnosus* GG. The strains in cluster A have sequence similarity at nucleic acid level to *L*. *rhamnosus* GG between 99.942 and 99.984% whereas the strains in cluster B differ remarkably more having the similarity between 97.0 and 98.5% (Fig 1). Interestingly, all the genomes of five isolated *L*. *rhamnosus* strains (Lrh13, Lrh22, Lrh28, Lrh30 and Lrh47) that contain plasmids belong to the cluster B (Table 2, Fig 1). The presence of unique orthologs between clusters were compared (S1 Table). Most of the differing orthologs that could be annotated, and are not phage sequences, are exopolysaccharide-related. Cluster A consists of 66 orthologs that were not found from cluster B and 35 orthologs that were missing from cluster A but present in cluster B. Interestingly, there were 8 EPS related ORFs present and 10 absent in cluster A.

### Complement activation and binding of complement regulators

**Fig 2:**
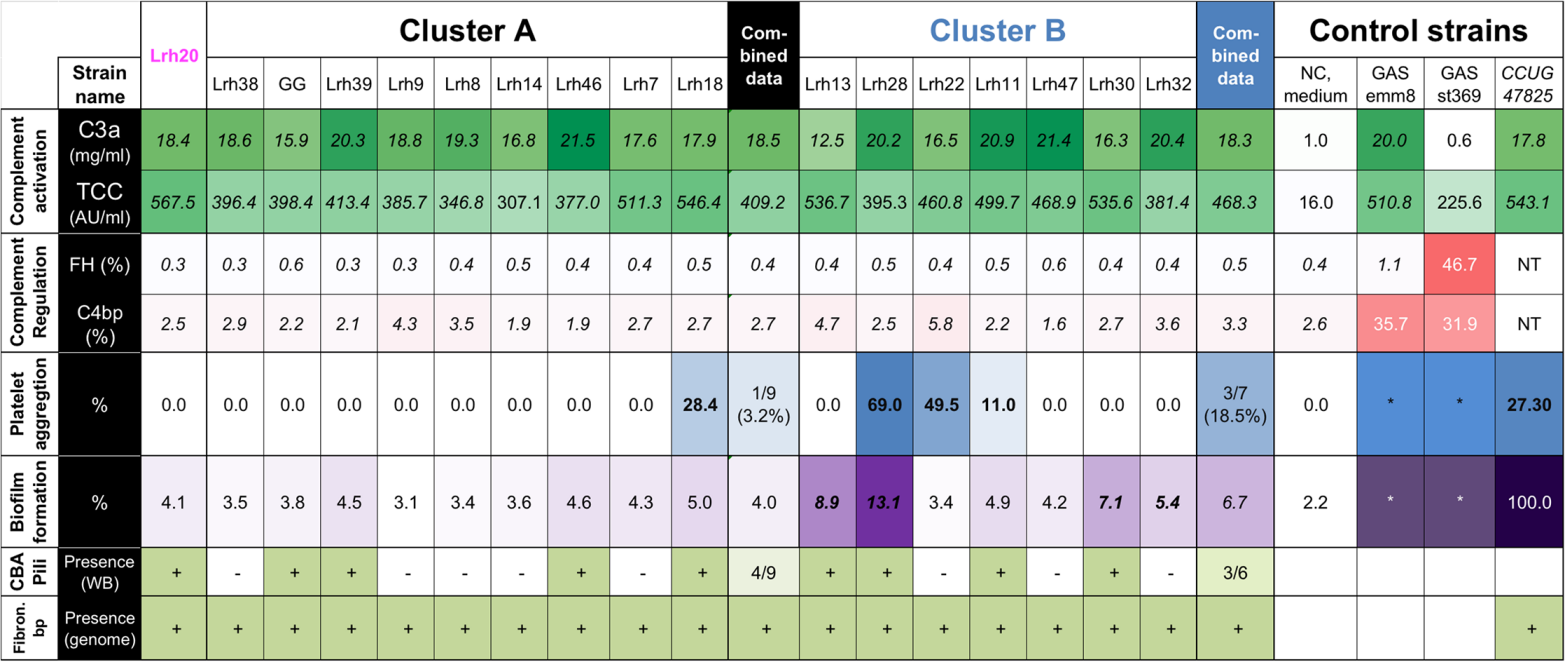
A heatmap of phenotypic features of the *L*. *rhamnosus* strains measured *in vitro*. Combined data represent average values or numbers of the strains detected in clusters A and B. NC equals negative control containing buffer in the assays, GAS group A streptococcus, NT not tested, asterisk* equals values that are representative from literature but not tested here for platelet aggregation [36] and biofilm formation [37], TCC equals terminal complement complex, FH factor H, C4bp complement C4b binding protein, CBA SpaC, SpaB and SpaA pilin subunits and fibron. bp equals fibronectin/fibrinogen binding protein. ***Bold and Italic letters*** equal significant value, p<0.05 for biofilm formation compared to *L*. *rhamnosus* GG (GG). Color intensities indicate the level of the values: green for complement activation measured as C3a and TCC, red for binding of complement regulators, blue for platelet aggregation, purple for biofilm formation and light green express the presence of SpaCBA pilus determined by Western blot using anti-SpaA antibody and fibronectin/fibrinogen binding protein at genome.

The recovery of *L*. *rhamnosus* isolates from the blood of bacteremic patients raised questions about their survival within the human bloodstream and also their (possible) recognition or killing by the host immunological system. Initial experiments indicated *L*. *rhamnosus* GG and clinical isolates showed only little survival in blood (less than 31% survival after 3 hours at 37°C) in contrast to strain st369, a known pathogen belonging to the Group A Streptococci, which multiplies rapidly in blood (at least 5 cell divisions after 3 hours at 37°C; see S2 Table). To further explore these, we studied *in vitro* if the isolated strains can activate the complement system and thus, be recognized by the human innate immune system, and if they are able to bind complement regulators to evade complement in the manner of pathogenic bacteria. Strains were incubated with human serum to trigger the complement activation response. All the isolates as well as *L*. *rhamnosus* GG induced the formation of C3a and soluble terminal component TCC in serum (Fig 2 and S3 Table). All tested *L*. *rhamnosus* strains induced significantly higher C3a concentrations in serum than the negative control (*S*. *pyogenes* st369*)*. Similar observations were made for the levels of TCC, although more variations were seen among isolates (notably between Lrh14 (the lowest) and Lrh7, Lrh13, Lrh18, Lrh20 and Lrh30 (the highest values)). Taken together, the amounts of both complement activity markers (C3a and TCC), indicated that the complement system was activated in the presence of the isolated *L*. *rhamnosus* strains *in vitro*, as opposed to the *S*. *pyogenes* st369, which did not activate the complement system similarly as earlier described [24].

To further explore additional factors mediating activation of the complement and to assess the ability of these strains to potentially inhibit complement activation, we examined the direct binding of complement regulators FH and C4bp to all *L*. *rhamnosus* strains (Fig 2, S3 Table). No binding of FH or C4bp to any of the *L*. *rhamnosus* strains was observed. The FH binding ranged between 0.3–0.55% and the C4bp binding between 1.6–5.8%, which corresponds to background levels (0.40 ± 0.15% and 3.1 ± 0.6%, respectively). In contrast, the control strain *S*. *pyogenes* st369 showed strong FH binding (46.7 ± 14.1%) and both *S*. *pyogenes* emm8 (35.7 ± 0.2%) and st369 (31.9 ± 14.7%) exhibited strong C4bp binding as previously reported [33]. These results indicate that the *L*. *rhamnosus* blood isolates and the probiotic strain GG do not bind these complement regulators and therefore do not escape complement using studied mechanisms as known in pathogens [38, 39].

### Human platelet aggregation by the *L*. *rhamnosus* strains

Next we studied the capacity of the *L*. *rhamnosus* strains to promote platelet aggregation. Platelets are cell fragments that plug the leakage in a damaged blood vessel but they are also key components in the innate immune system [40]. Many Gram-positive bacteria causing sepsis have been shown to induce platelet aggregation [41]. The *L*. *rhamnosus* strains were tested for platelet aggregation using isolated platelets from different human donors (Fig 2, S3 Table). Four out of 16 *L*. *rhamnosus* isolates (Lrh11, Lrh18, Lrh22, Lrh28) induced platelet aggregation. It is noteworthy that platelet aggregation was donor-dependent. Hence the same strain did not always induce platelet aggregation for every donor tested and this was also noted for the positive control *L*. *salivarius* CCUG 47825 (S3 Table). Two of the aggregating strains, Lrh22 and Lrh28, induced platelet aggregation with platelets from every donor tested. Similar to previous studies [17] *L*. *rhamnosus* GG and the rest of the twelve isolates did not induce platelet aggregation for any tested donors. The experimental data shown here suggest that the *L*. *rhamnosus* blood isolates differ in their ability to cause platelet aggregation and that platelet aggregation was not a common phenotypic trait associated with *L*. *rhamnosus* blood isolates although 2 clinical strains were highly proficient platelet aggregators.

### Biofilm formation by the *L*. *rhamnosus* strains

Biofilm formation is a feature found in both pathogenic and commensal bacteria, as it promotes colonization and persistence in an ecological habitat. Hence, we assessed the formation of biofilms for *L*. *rhamnosus* strains in 24-well plates (Fig 2 and S3 Table). Some differences in biofilm formation could be seen between the isolated strains. Four of the strains (from cluster B) tested (Lrh13 (8.9 ± 3.1%), Lrh28 (13.1 ± 4.7%), Lrh30 (7.1 ± 2.3%), Lrh32 (5.4 ± 1.3%)) showed a stronger ability for biofilm formation than GG (3.8 ± 0.8%). *L*. *salivarius* CCUG 47825 was used as a positive control and produced a strong biofilm (relative biofilm formation 100 ± 25.7%). Summarizing, biofilm formation varied between *L*. *rhamnosus* isolates with some strains displaying higher biofilm formation than *L*. *rhamnosus* GG. All those strains that showed clear biofilm formation phenotype belong to cluster B.

### Comparison of LPXTG protein encoding genes, SpaCBA pilus proteins and EPS related genes

In an attempt to further characterize the phenotypes of these different isolate, we examined some of the relevant genomic features related to surface exposed structures and molecules of these strains. Typically, the genomes of Gram-positive bacteria harbor genes that code for LPXTG proteins as well as the sortase enzymes. Sortases are involved in the surface display and cell wall anchoring of surface proteins carrying the sortase-specific recognition motif LPXTG in Gram-positive bacteria [42]. Based on the genome sequences of the blood isolates, we investigated the repertoire of all gene sequences that are predicted to code for LPXTG proteins, as they are most likely to interact with the environment and the host, in that particular context with the human vascular system. All strains possessed a distinct and unique set of proteins (S2 Fig). It has been well established that the presence of pili depends on a specific set of LPXTG proteins that include major pilin, minor pilin and, tip pilin subunit proteins and these are widely spread in various *Lactobacillus* spp. [2]. In *L*. *rhamnosus* GG, two pili gene clusters have been identified but only SpaCBA pili have been found to be produced [22, 43]. Hence, we used Western blot analysis with SpaA and SpaD antibodies to show that 8 out of 16 *L*. *rhamnosus* strains produced SpaCBA pili while none were producing the SpaFED pili (Fig 2) even though the SpaFED locus was found in all strains (S1 Fig). Five of these showed phenotypic characters (platelet aggregation (Lrh18, Lrh11) or biofilm formation (Lrh13, Lrh30) or both (Lrh28; Fig 2)). When the strains were divided into two groups based on the presence of SpaCBA pilus there was a significant association between the biofilm formation and the presence of pilus among the isolated strains (6.5 ± 3.11% (present) vs 3.87 ± 0.72% (absent), *p = 0*.*047*, S3 Table).

**Fig 3:**
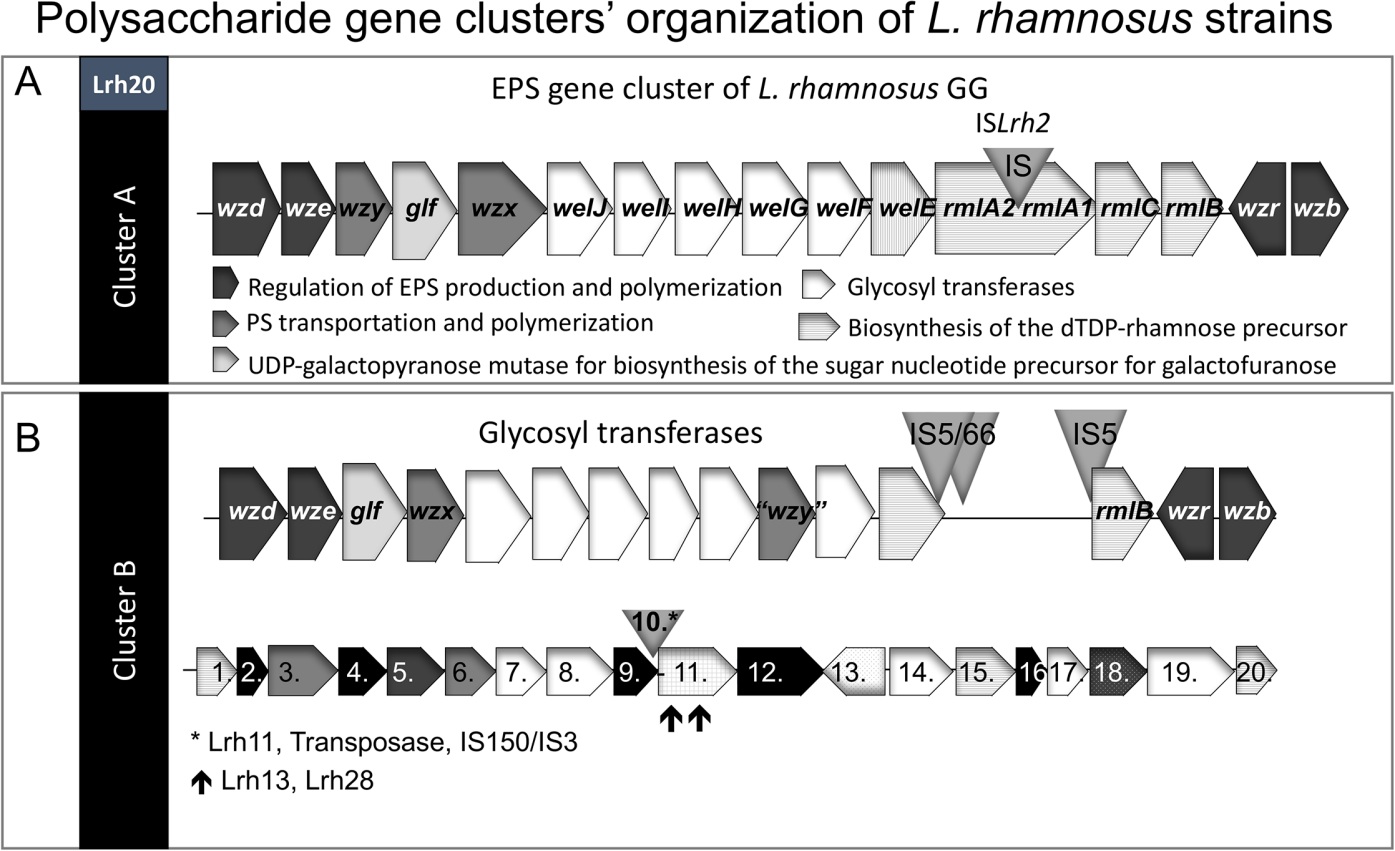
Genomic organization of polysaccharide clusters in *L*. *rhamnosus* strains. A) EPS gene cluster of *L*. *rhamnosus* GG is found from *L*. *rhamnosus* strains of cluster A and strain Lrh20. Insertion sequence (IS)-like elements differ between the strains. B) The *L*. *rhamnosus* strains of cluster B have differences at sequences encoding genes in the EPS gene cluster compared to *L*. *rhamnosus* GG. The genes similar to the EPS gene cluster of *L*. *rhamnosus* GG are named in the schematic illustration in the upper part (B). These regions include multiple genes coding IS-like elements such as IS5, IS66 and DDE transposases in the cluster B. The strains in cluster B have also other type of EPS/CPS gene cluster based on BLAST annotation results shown at the bottom (B). It consists of 19 or 20 genes which are annotated as 1. peptidoglycan N-acetylglucosamine deacetylase (EC 3.5.1.104), 2. hypothetical protein (FIG00750667), 3. capsular polysaccharide biosynthesis protein, 4. hypothetical protein (FIG00754398), 5. tyrosine-protein kinase transmembrane modulator EpsC, 6. putative O-unit flippase, 7. glycosyl transferase, group 0, 8. glycosyl transferase family protein, 9. hypothetical protein (FIG00752018), 10. mobile element protein, 11. lysozyme M1 (1,4-beta-N-acetylmuramidase), 12. hypothetical protein (FIG00747485), 13. uncharacterized conserved protein, similar to IcaC of Staphylococcus; YHJR B.subtilis family, 14. glycosyltransferase, 15. UDP-N-acetylglucosamine 2-epimerase (EC 5.1.3.14), 16. hypothetical protein (FIG00750997), 17. lipopolysaccharide synthesis sugar transferase, 18. polysaccharide biosynthesis protein CpsM, 19. putative N-acetylgalactosaminyldiphosphoundecaprenol and 20. UDP-glucose 4-epimerase (EC 5.1.3.2). * = Transposase, IS150/IS3 present (only in Lrh11), ↑ = site of point mutations in the gene encoding lysozyme M1(1,4-beta-N-acetylmuramidase) (present in Lrh13 and Lrh28). The illustration of EPS cluster in 3A is modified after [46].

As the surface polysaccharides are known to participate in important functions during pathogenesis, such as tissue adherence, biofilm formation and evasion of host defence [44, 45] and our clusters of *L*. *rhamnosus* strains showed differences between the ORFs related to EPS/CPS gene clusters (S1 Table), we wanted to study if the genomic organization of EPS gene clusters in the studied blood isolates of *L*. *rhamnosus* are similar to *L*. *rhamnosus* GG [46]. This cluster consists of genes coding proteins for the regulation of EPS production and polymerization (*wzd*, *wze*, *wzr*, *wzb*), polysaccharide transportation and polymerization (*wzx*, *wzy*), glycosyltransferases (*welJ*, *welI*, *welH*, *welG*, *welF*, *welE*), biosynthesis of the dTDP-rhamnose precursor (*rmlA*, *rmlC*, *rmlB*) and UDP-galactopyranose mutase for biosynthesis of the sugar nucleotide precursor for galactofuranose (*glf*) [46]. Interestingly, after annotation analysis our data showed that the strains in cluster A had the EPS cluster consisting 16 putative ORFs/genes with similar identity (> 78%) to those present in *L*. *rhamnosus GG* (Fig 3A). However, the strains in cluster B had only some of these genes (*wzd*, *wze*, *wzr*, *wzb* and *rmlB* (except Lrh22)) with similar identity (> 78%) to genes in EPS cluster of *L*. *rhamnosus* GG (Fig 3B). The other genes found in this cluster were annotated to encode genes for mostly glycosyl transferases with different specificities, hypothetical proteins and several mobile element proteins. However, we were not able to characterize these clusters in details because multiple mobile elements were present and they spliced the sequences into multiple contigs. Interestingly, the strains in cluster B consist also a different type of polysaccharide (EPS/CPS) cluster comprising of 19 genes (Fig 3B) including an additional flippase (6.) gene essential for EPS production. The cluster was similar in most of the strains (Lrh22, Lrh30, Lrh32 and Lrh47) in the cluster B (Fig 3B). The strain Lrh11 had a mobile element in the PS cluster before the gene coding for lysozyme and the strains Lrh13 and Lrh28 had two point mutations within the lysozyme gene (resulting in a split gene). The differences in the organization and existence of the polysaccharide clusters may have an influence on the polysaccharide surface of the strains and could potentially lead differences in the phenotype of these isolates.

## Discussion

**Fig 4:**
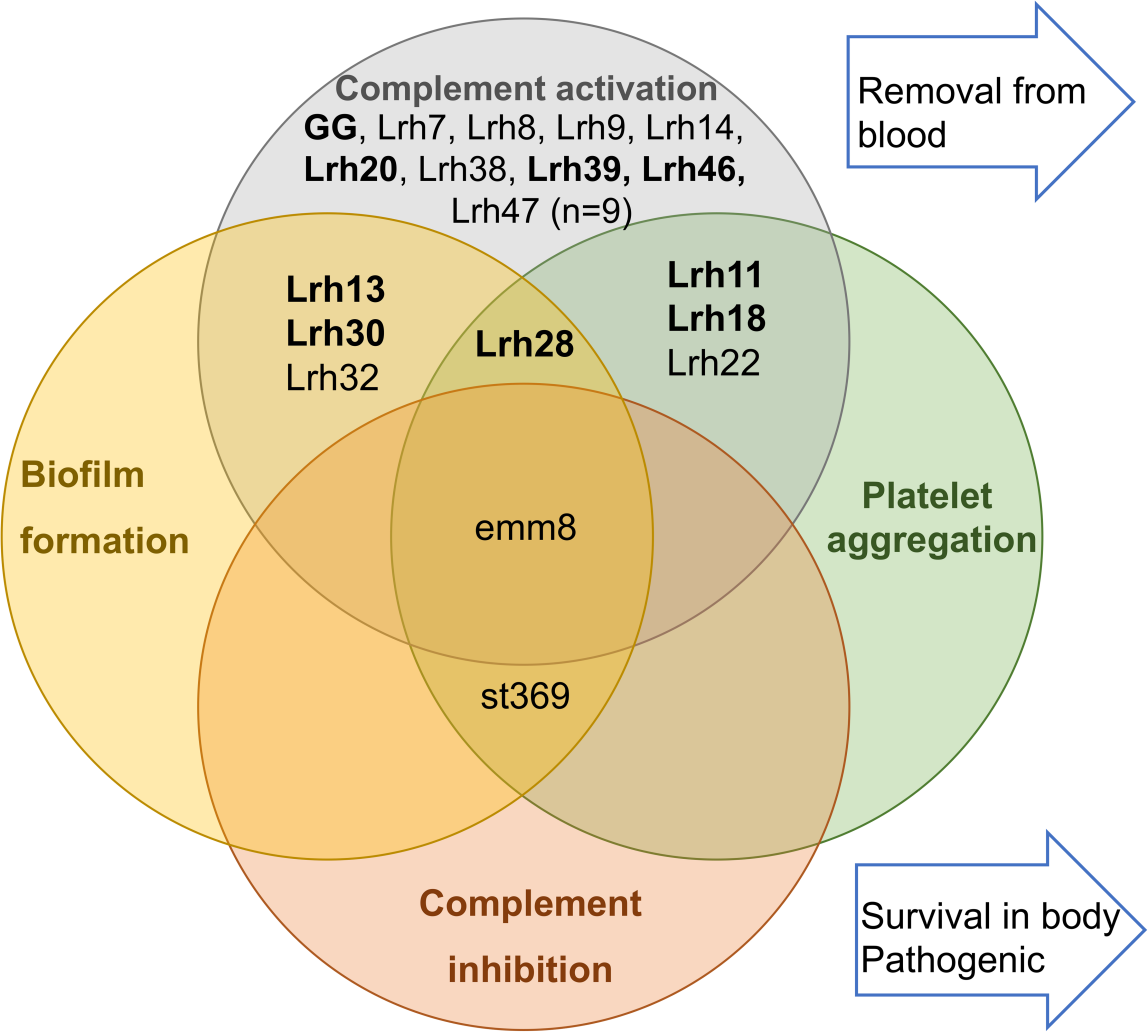
Immunological properties of *L*. *rhamnosus* GG, clinical isolates and pathogens towards the human host based on *in vitro* data. Strains Lrh7, Lrh8, Lrh9, Lrh14, Lrh20, Lrh46, Lrh47, Lrh38 and Lrh39 displayed comparable properties as observed in *L*. *rhamnosus* GG. All these strains activate the complement in serum. Strains Lrh13, Lrh30 and Lrh32 also induced more biofilm formation more than GG. Strains Lrh11, Lrh18 and Lrh22 were aggregating with platelets. Strain Lrh28 was doing both causing platelet aggregation and biofilm formation. The pathogenic group A *Streptococcus* strain emm8 induced complement activation, but it could bind complement regulator C4bp to evade complement. The pathogenic strain st369 could bind complement regulators C4bp and factor H and did not induce complement activation. A similar type of GAS strain has been shown to cause platelet aggregation [36] and biofilm formation [37]. The bolded strains have SpaCBA pili similar to GG.

Although *Lactobacillus* spp. are commensals, part of the normal flora of the gastrointestinal tract, oral cavity and female urogenital tract [47], they have been occasionally isolated from immunocompromised and other hospitalized patients [6]. In the present study, we have isolated and sequenced 4 new strains and 12 already described *L*. *rhamnosus* strains from blood cultures collected from bacteremic patients between years 2005 and 2011 in HUSLAB. The main purpose of this study was to first identify the isolated strains at the sequence level and compare them to the widely consumed probiotic *L*. *rhamnosus* GG and, secondly, to investigate the phenotypic features related to complement evasion, platelet aggregation and biofilm formation in these isolated *L*. *rhamnosus* strains and compare these characteristics with *L*. *rhamnosus* GG. Our data revealed that *L*. *rhamnosus* isolates were clearly different from *L*. *rhamnosus* GG and each other at sequence level and they did not possess any distinctive niche specific (blood) genotypic traits as compared to *L*. *rhamnosus* GG. The strains were clustered into two main clusters A and B. Cluster A includes *L*. *rhamnosus* GG and its related strains. A model summarizing our findings (Fig 4**)** shows that all the isolated *L*. *rhamnosus* strains, including *L*. *rhamnosus* GG, were able to activate the complement system when it was measured as C3a and TCC formation in serum. Similarly, the strains did not bind complement inhibitors C4bp or FH. These results indicate that *L*. *rhamnosus* strains have not acquired mechanisms or traits to escape the complement system as opposed to the tested pathogens. However, some differences were seen in other phenotypic features, since four of the sixteen strains induced platelet aggregation and four strains formed stronger biofilm compared to *L*. *rhamnosus* GG. Most of these strains belong to the cluster B. The *L*. *rhamnosus* blood isolates showed a high level of heterogeneity and showed no common phenotypic trait that is involved in the persistence in the host, like biofilm formation, platelet aggregation and specific pilus production. Interestingly, analysis of genes encoding polysaccharide clusters showed differences between the strains in cluster A and B that might be also influencing the differences seen in phenotypes.

Our results of the current study extend the results of the earlier survey by Salminen and co-workers [48] and unambiguously demonstrate that the widely consumed *L*. *rhamnosus* GG was not associated with the bacteremia cases. The techniques used for the analysis of the strains at sequence level have been highly improved from earlier used PFGE method to full genome sequence analyses up to 100% coverage of the sequence that allow to distinguish the strains at species level. However, there are reports that some cases of bacteremia have been associated with the consumption of probiotics among certain special conditions including hematopoietic stem cell transplantation [49], HIV-infection [50], severe active ulcerative colitis [13] and in a newborn with intrauterine growth restriction [51]. However, not all studies have determined the strains at species level. Only Sadowska-Krawczenko (2014) analyzed the identity at 100% (16S sequencing and PCR) in the case of intrauterine growth restriction [51]. A case report demonstrates that it is important to use the molecular diagnostic tools that can distinguish the strains at species level when we want study if the blood isolate originates from the probiotic strain consumed [52]. It is known earlier that immuno-compromised patients and patients with underlying diseases such as cancer are the ones who usually suffer from bacteremia caused by lactobacillus [48, 53]. Based on our and others’ studies we conclude that healthy people can use probiotics safely but among risk patients having disturbed immune function and mucosal disruption as in the case of inflammatory bowel diseases, the consumption of probiotics should be considered with caution before new studies have carefully assessed the risk to these patients.

All the *Lactobacillus* strains were recognized by the complement system and had no properties to inhibit the complement system by binding complement regulators (C4bp or FH). Thus, in this respect they can be categorized as commensals in contrast to pathogenic GAS strains emm8 and st369 that we used in this and an earlier study [24]. However, some variation in the level of TCC formation between the strains could be seen. Recently, piliation of *L*. *rhamnosus* GG has been shown to bind macrophages through a pattern recognition receptor namely complement receptor type 3 (CR3, iC3b receptor, CD11b/18) that mediates phagocytosis and removal of bacteria [54]. The SpaCBA piliation was also analyzed in the current study (Fig 2). The group having pili showed to have only a borderline increase in the complement TCC level (483.9 ± 76.4 AU/ml and 407.3 ± 68.1 AU/ml, p = 0.053; S2 Table) compared to the group without the SpaCBA pili. However, when the difference in complement activation as TCC between the SpaCBA pilus-deficient mutant strain of *L*. *rhamnosus* (PB12) [55] and *L*. *rhamnosus* GG was tested no significant difference was seen (441.5 ± 78.4 AU/ml vs 398.4 ± 87.1 AU/ml, p = 0.07, respectively). Both of these strains induced similarly TCC formation in serum indicating that pilus as a structure is not inducing more complement activation. Thus, our data suggest that the isolated strains are recognized by the complement and they do not differ from each other in the ability to induce complement activation in serum and have not developed properties similar to pathogens.

Moreover, in our study we demonstrated that some of the strains have phenotypic features that are different from *L*. *rhamnosus* GG but can be found from pathogenic strains. These phenotypic features were abilities to cause platelet aggregation and biofilm formation. Earlier studies with a handful of strains have also shown that some of the clinical isolates of *L*. *rhamnosus* have been able to cause platelet aggregation [16, 18]. Those strains were isolated from infectious endocarditis (5/5 tested strains), laboratory strains (8/16 strains) [15] and strains from infection of aortic aneurysm graft and carcinoma with liver metastasis [18]. Similar to our findings, *L*. *rhamnosus* GG did not cause aggregation with platelets in previous studies [17, 18]. The ability to bind fibrinogen is known to help Gram-positive pathogens escape the immune system, and can be sufficient to induce platelet aggregation which can lead to e.g. endocarditis [41]. Recently a *L*. *salivarius* isolate from a case of sepsis, was found to aggregate human platelets via binding human fibrinogen through a novel fibrinogen-binding protein [19]. In our study a gene encoding fibrinogen/fibronectin binding proteins was present but we could not show that it causes the platelet aggregation phenotype (Fig 3).

We found that five of the isolated *L*. *rhamnosus* strains (Lrh28, Lrh13, Lrh30, Lrh32) were able to form biofilm more than *L*. *rhamnosus* GG (Fig 2). Interestingly, one strain (Lrh28) had the platelet aggregation phenotype, as well. Biofilms of lactobacilli can be located in many natural environments. Pathogenic bacteria can utilize the biofilm formation capacity for better survival in human body [56]. Various genetic pathways including clusters producing surface polysaccharides (EPS) have been shown to influence the biofilm formation capacity of *L*. *rhamnosus* GG in an earlier study [46]. Similar to a previous study we found in our experiments that *L*. *rhamnosus* GG was able to form small amounts of biofilms in the MRS medium [57]. Interestingly, we found that the strains in cluster A and cluster B possessed different EPS clusters so that the strains in cluster A had the EPS cluster similar to *L*. *rhamnosus* GG but the strains in cluster B had different sets of genes encoding proteins needed for EPS/CPS production such as glycosyltransferases and hypothetical proteins than GG. They also had an additional polysaccharide cluster that was found from all those strains in cluster B. However, one of the limitations of our study is that the genomic sequences of the isolated *L*.*rhamnosus* strains were not complete and contained some gaps. Moreover, we did not compare the gene expression of the *L*. *rhamnosus* strains and hence could not correlate all our functional analyses to the gene level. At least partly some of the phenotypic features could be caused by differences in the organization of EPS/CPS clusters. Recently, it has been shown that alterations in the genes involved in EPS synthesis in *Lactobacillus johnsonii* changed their surface properties and influenced on biofilm formation, cell adhesion, and autoaggregation [58]. Two clinical *L*. *rhamnosus* strains isolated from dental pulp infections have been shown to have modified exopolysaccharide clusters [21]. Such a biofilm producing phenotype can provoke the development of infections e.g. in the case of dental pulps, during catheter usage or endocarditis and be detrimental to a patient during bacteremia. However, biofilm formation is usually a feature associated with niche colonization in a different context than in clinical cases.

To conclude, our data demonstrate that *L*. *rhamnosus* strains isolated from blood cultures are distinct from *L*. *rhamnosus* GG and well-recognized by the body through the complement system and differ clearly from pathogenic strains. This supports the safe use of *L*. *rhamnosus* GG in healthy subjects with a functional immune system. Clinical blood isolates of *L*. *rhamnosus* do not possess any distinctive niche-specific genotypic traits for the blood environment similar to pathogens, which may be explained by the fact that they resided for a short period of time in blood and originally derived from other niches such as mouth, vagina, intestine or food products. In that regard, some of the strains that have platelet aggregation and biofilm formation properties might originate from those types of niches, where these properties are central for the stability and vitality of the strain.

## Supporting information

S1 Table Orthologs uniquely present and absent in Cluster A compared to Cluster B. (XLS)

S2 Table Blood survival assay. Experiments showing the survival of strains *L*. *rhamnosus* GG, clinical blood isolate of *L*. *rhamnosus* and st369 a group A streptococcus in blood.
(XLSX)

S3 Table The original data for phenotypic features of the *L*. *rhamnosus* strains measured *in vitro*. The data consist of complement C3a and TCC formation after stimulation with the strains, the binding of complement inhibitors factor H and C4bp and determination of platelet aggregation and biofilm formation.
(XLSX)

S1 Fig Western blots of the cell wall protein extracts detected with SpaA or SpaD antibodies. Polyclonal (Rabbit) anti-SpaA and anti-SpaD were used as 1:10000 dilution. Molecular masses (kDa) of the standard proteins are depicted on the left side of the blots. Lr GG equals *L*. *rhamnosus* GG. The methods are described in detail [22].
(PDF)

S2 Fig LPXTG genes ordered based on a dendrogram illustrating the phylogeny of *Lactobacillus rhamnosus* strains. Platelet aggregation and biofilm formation data (similar to Fig 2) are shown. LPXTG genes were searched based on the known LPXTG sequences shown. Six of them were present only in cluster B.
(PPTX)
