# Correction: Genotypic and phenotypic diversity of *Lactobacillus rhamnosus* clinical isolates, their comparison with strain GG and their recognition by complement system

**DOI:** 10.1371/journal.pone.0181292

**Published:** 2017-07-10

**Authors:** Eija Nissilä, François P. Douillard, Jarmo Ritari, Lars Paulin, Hanna M. Järvinen, Pia Rasinkangas, Karita Haapasalo, Seppo Meri, Hanna Jarva, Willem M. de Vos

Table 1 appears incorrectly. Please see the complete, correct Table 1 here.

**Table 1:** Genetic information of *L*. *rhamnosus* strains isolated from blood cultures in HUSLAB, 2005–2011.

Strain name	Isolated from	Length (Mb)	G+C (%)	ORFs	Contigs	Coverage	Accession number	Reference
*L*. *rhamnosus* GG (ATCC 53103)	healthy intestine	3.01	46.7	2926	1	Full coverage	NC_013198	[29]
Lrh7	blood	2.86	46.7	2851	86	17	JTHS00000000	[30]
Lrh8	blood	2.86	46.7	2875	86	17	JTHR00000000	[30]
Lrh9	blood	2.88	46.7	2864	112	13	JTHQ00000000	[30]
Lrh11	blood	2.89	46.6	2923	112	10	JTIT00000000	[30]
Lrh13	blood	2.9	46.7	2891	209	9	JTIR00000000	[30]
Lrh14	blood	2.9	46.6	2883	260	9	JTIQ00000000	[30]
Lrh18	blood	2.93	46.6	2918	222	9	JTIM00000000	[30]
Lrh20	blood	2.93	46.6	2929	102	16	JTIJ00000000	[30]
Lrh22	blood	2.96	46.6	2988	195	9	JTIH00000000	[30]
Lrh28	blood	3.00	46.6	2982	112	19	JTIB00000000	[30]
Lrh30	blood	3.01	46.6	3010	96	12	JTHY00000000	[30]
Lrh32	blood	2.95	46.6	2980	64	18	JTHW00000000	[30]
Lrh38	blood	2.88	46.7	2648	66	27	LPNU00000000	In this study
Lrh39	blood	3.00	46.7	2879	62	45	LPNT00000000	In this study
Lrh46	blood	3.09	46.7	2868	71	19	LPNW00000000	In this study
Lrh47	blood	3.08	46.8	2860	51	14	LPNV00000000	In this study
