# Supplementary material for: Genotypic and phenotypic diversity of Lactobacillus rhamnosus clinical isolates, their comparison with strain GG and their recognition by complement system
Source: PLoS One. 2017 May 11;12(5):e0176739. doi: 10.1371/journal.pone.0176739 (PMC5426626; doi:10.1371/journal.pone.0176739)

Detection of SpaA and SpaD proteins *on L. rhamnosus* strains by Western Blots

Methods:
Previously described [29](Kankainen et al. 2009)
The methods includes extraction of cell wall proteins,
SDS-PAGE and detection of SpaA and SpaD on membrane
using polyclonal (rabbit) anti-SpaA or anti-SpaD antibodies (1:10000 dilution) and
development of the films using ECL kit.

S1 Fig A: Western blots detecting Anti-SpaA

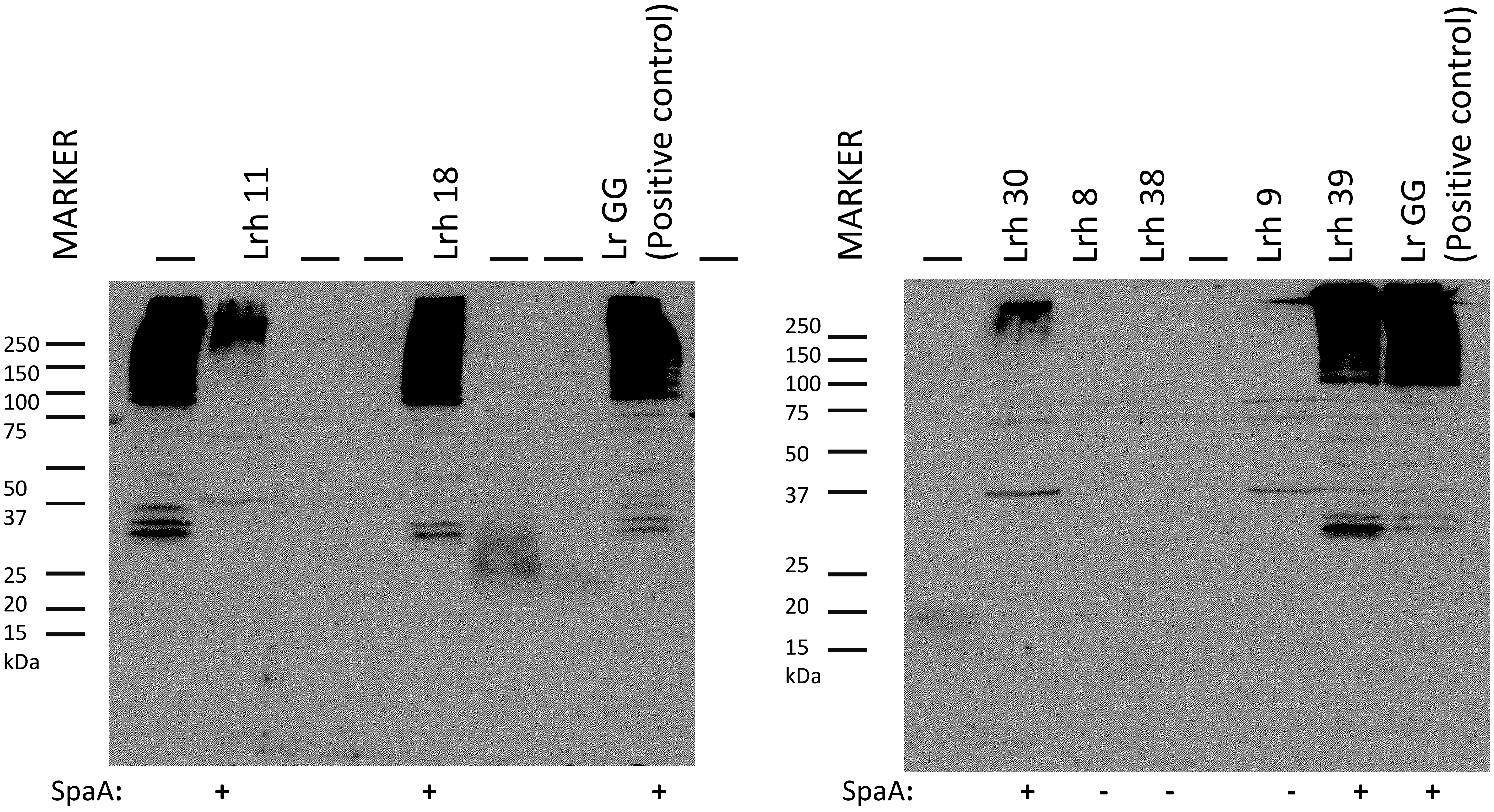

S1 Fig B: Western blots detecting Anti-SpaA

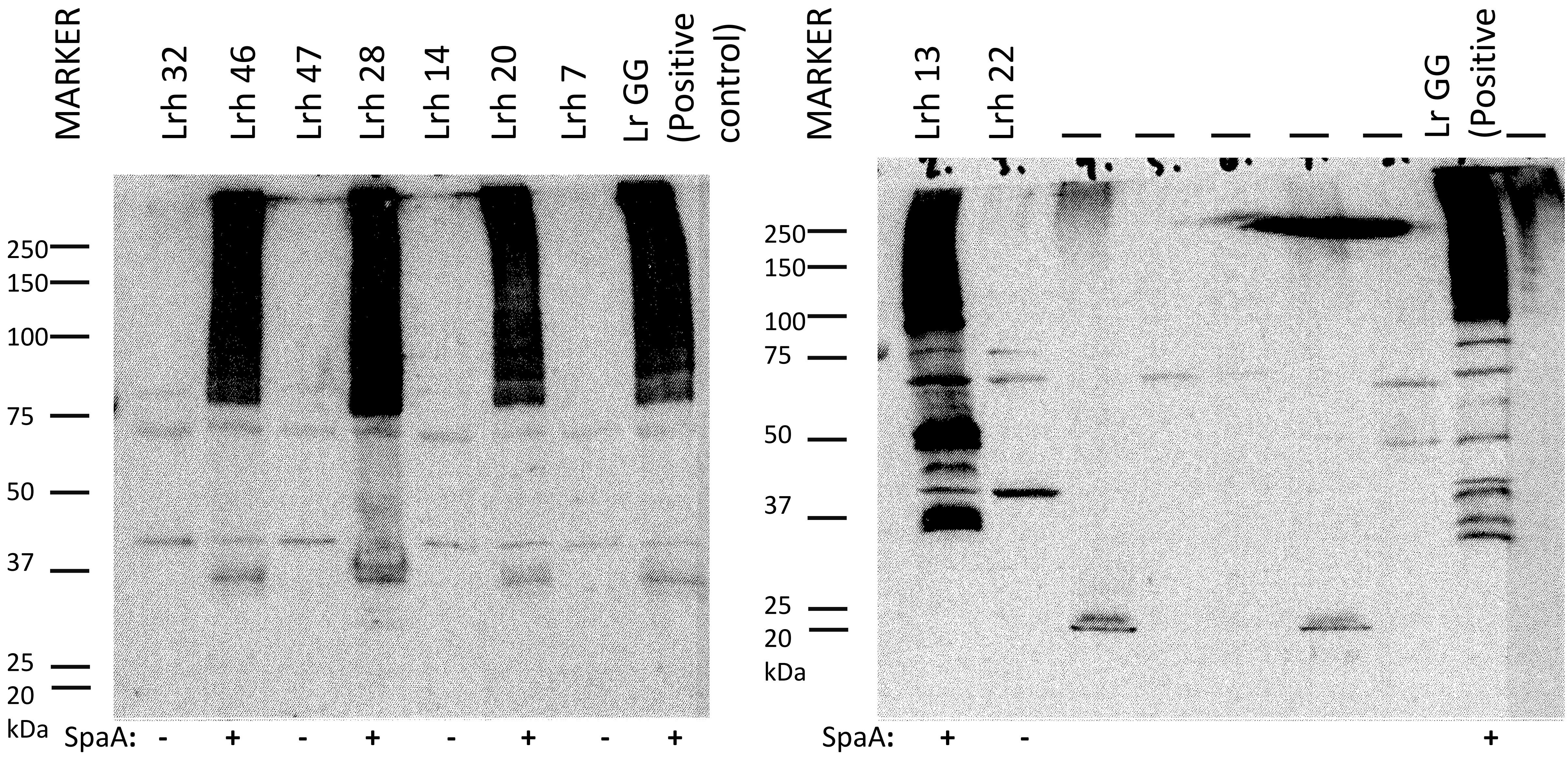

S1 Fig C: Western blots detecting Anti-SpaD

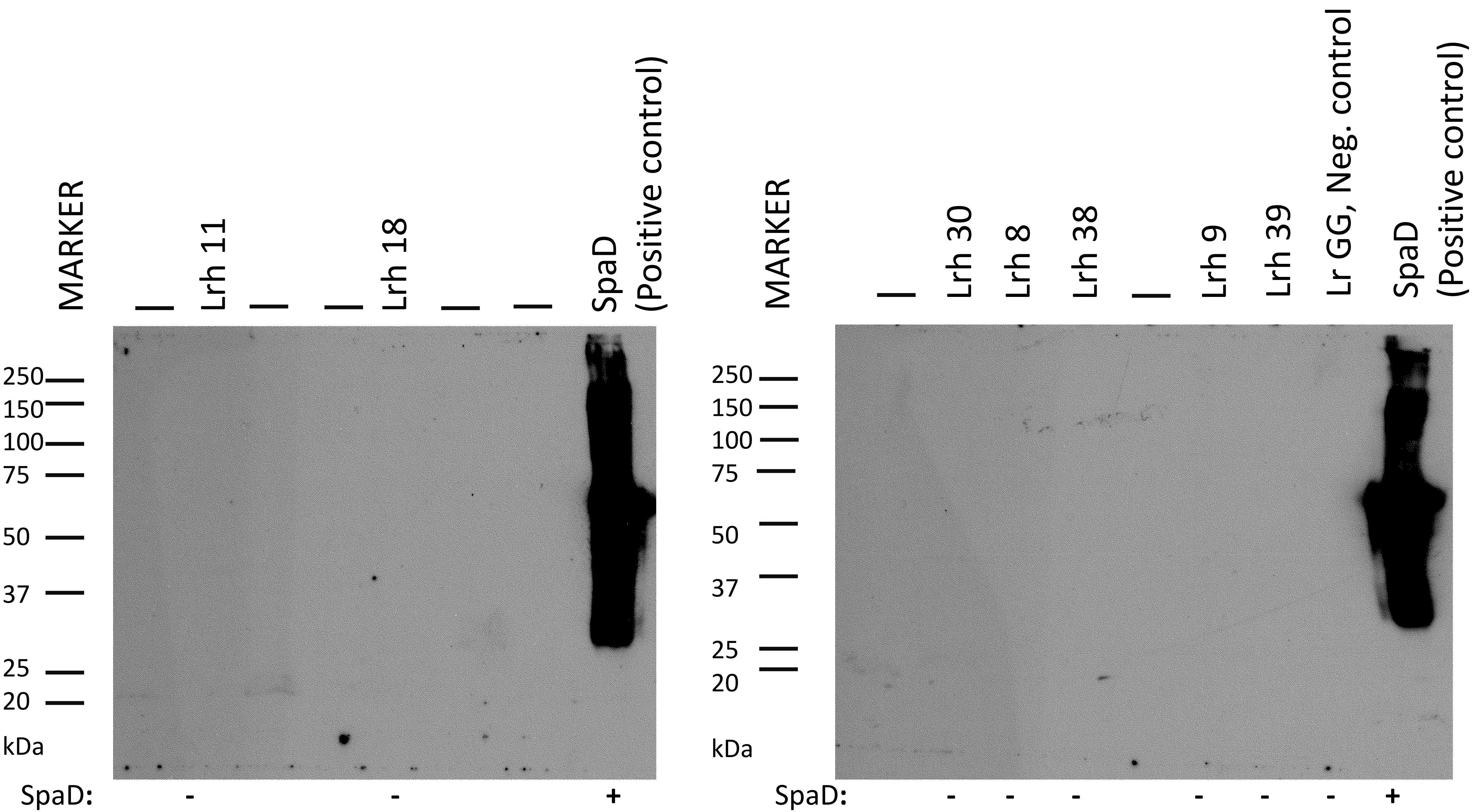

S1 Fig D: Western blots detecting Anti-SpaD

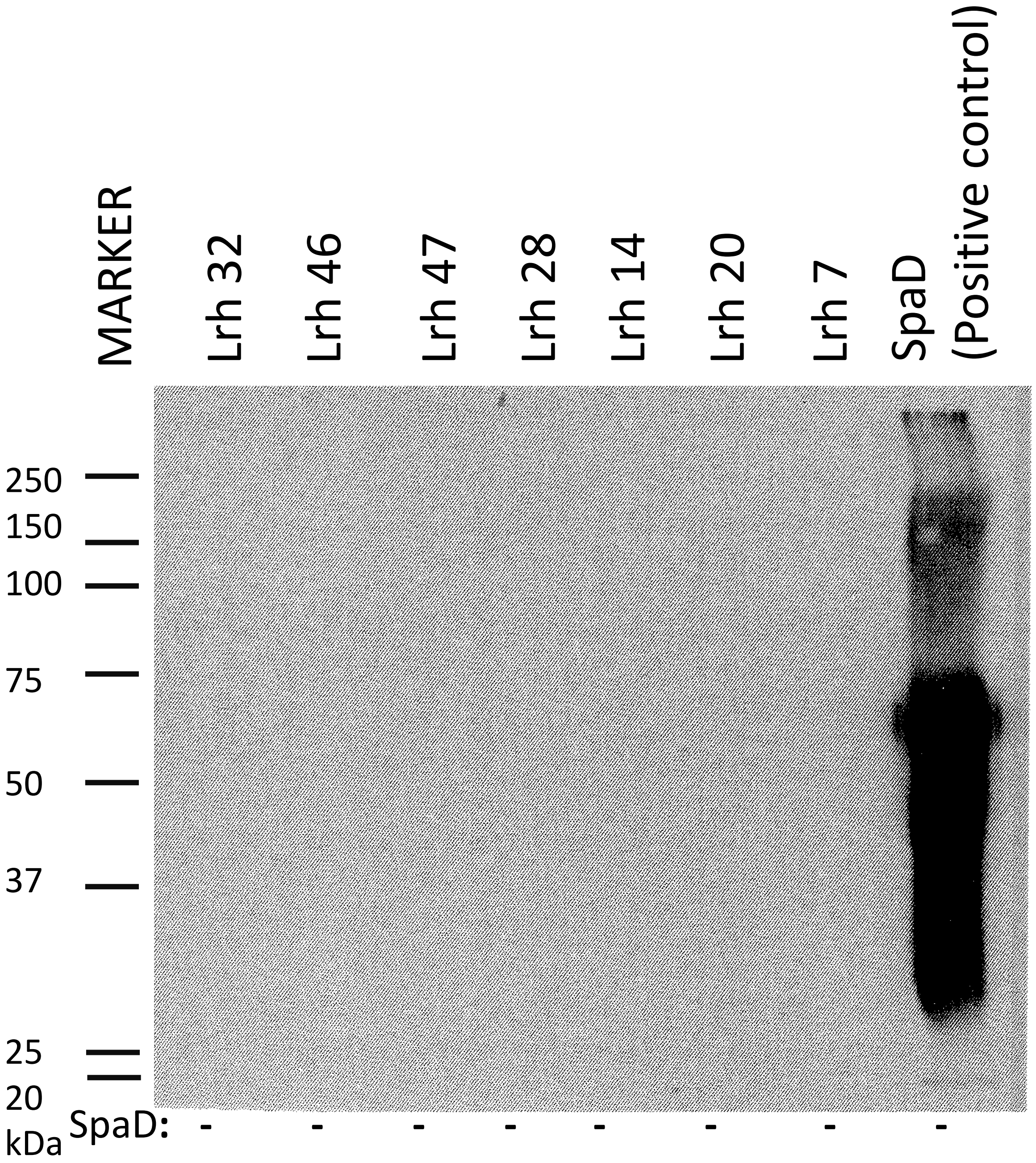

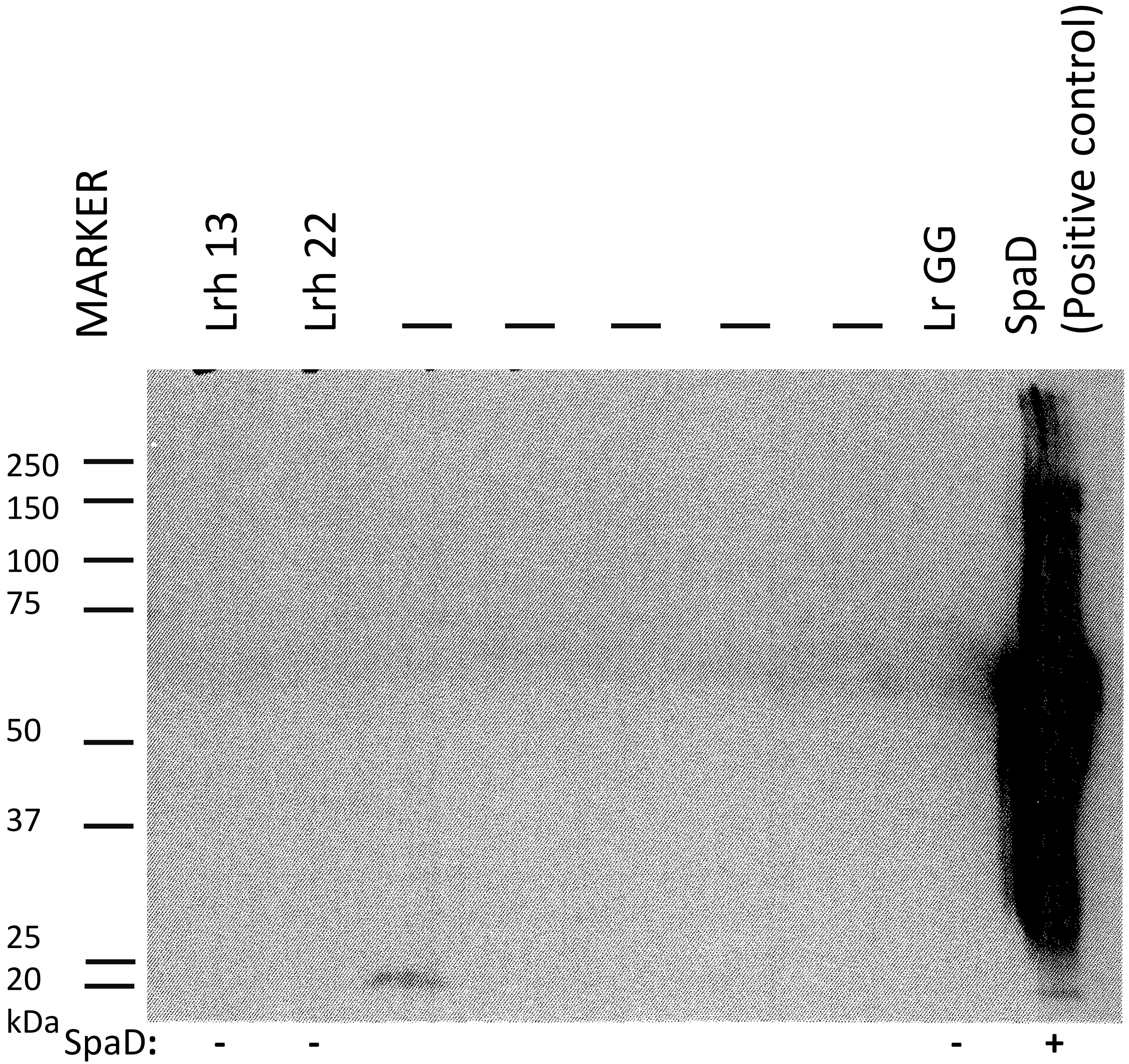

Supplement: S1 Fig — Polyclonal (Rabbit) anti-SpaA and anti-SpaD were used as 1:10000 dilution. Molecular masses (kDa) of the standard proteins are depicted on the left side of the blots. Lr GG equals L. rhamnosus GG. The methods are described in detail [22]. (PDF) [file pone.0176739.s004.pdf]
