# Supplementary figures and images for: Genotypic and phenotypic diversity of Lactobacillus rhamnosus clinical isolates, their comparison with strain GG and their recognition by complement system
Source: PLoS One. 2017 May 11;12(5):e0176739. doi: 10.1371/journal.pone.0176739 (PMC5426626; doi:10.1371/journal.pone.0176739)

## Slide 1
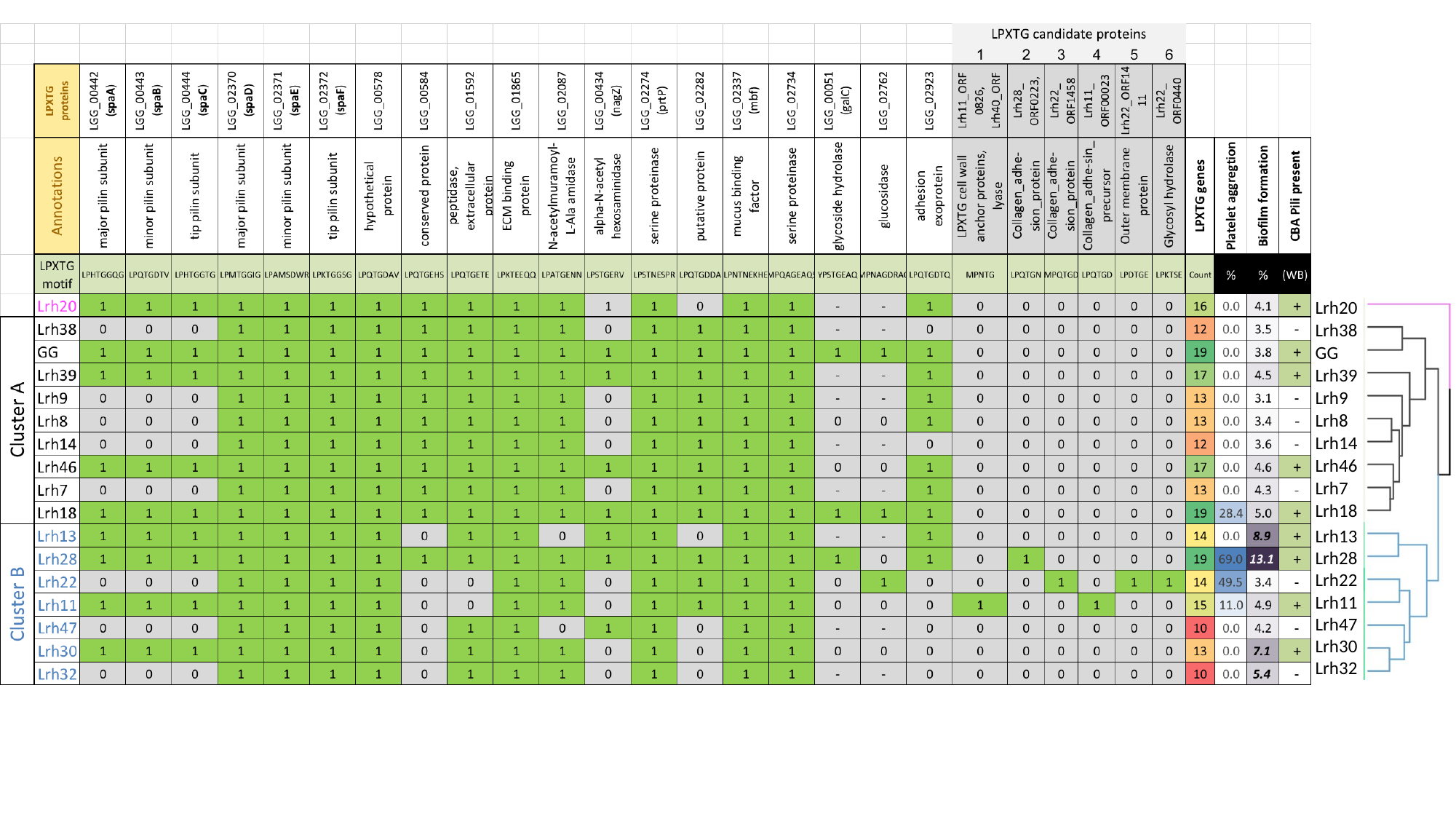

Lrh20
Lrh38
GG
Lrh39
Lrh9
Lrh8
Lrh14
Lrh46
Lrh7
Lrh18
Lrh13
Lrh28
Lrh22
Lrh11
Lrh47
Lrh30
Lrh32

Supplement: S2 Fig — Platelet aggregation and biofilm formation data (similar to Fig 2) are shown. LPXTG genes were searched based on the known LPXTG sequences shown. Six of them were present only in cluster B. (PPTX) [file pone.0176739.s005.pptx]
